# Chicago Gynecological Society

**Published:** 1886-01

**Authors:** 

**Affiliations:** 2330 Indiana Avenue


TRANSACTIONS OF THE CHICAGO GYNÆCOLOGICAL SOCIETY.

*Regular Meeting, 27th November, 1885.* THE PRESIDENT, DANIEL T. NELSON, M. D., in the chair.

I. BARTLETT. *Remarks on the Toxic Properties of Sassafras.*

II. EARLE. *Remarks upon a Teratom, and a Case of Artificial Abortion*, with presentation of specimens.

I. DR. JOHN BARTLETT read a paper entitled,

REMARKS ON THE TOXIC PROPERTIES OF SASSAFRAS, from which it was apparent that the oil of sassafras exerts a decidedly toxic influence, although many standard works, consulted by DR. BARTLETT, make no allusion to any such quality in sassafras. DR. BARTLETT also cited instances in which "Sassafras-tea" had been used for its oxytocic and ecbolic effects.

He said his remarks had been largely based on a report made by DR. HILL, of Baltimore, of his observations and his experiments in the use of oil of sassafras, and he concluded his remarks by saying that "A study of the toxic effects of sassafras as reported by DR. HILL, and here suggested, would seem to show a triple resemblance to three familiar articles, opium, strychnine and ergot.

"In its action as a narcotic and sudorific it resembles opium.

"In its property of inducing tetanic and clonic spasms, followed by paralysis, it is similar to strychnine.

"In its power hinted at of exciting the uterus, it may be likened to ergot.

"It may be of interest here to call attention to the fact that the first reference to the use of ergot as an ecbolic was made by Stearns in 1807, whereas it had been used by midwives certainly as early as 1688, and probably very much earlier."

PROFESSOR JAMES H. ETHERIDGE referred to the action of the oil of sassafras on the motor centres in the spinal cord, supplying the uterus.

DR. EDWARD WARREN SAWYER said in New England, sassafras was a popular emmenagogue. Mothers were in the habit of giving decoctions of sassafras and tansy to their daughters in case of delayed or suppressed menstruation. Many of the essential oils produced the effects ascribed to sassafras by DR. BARTLETT. In the South, oil of sassafras was a popular remedy for uterine disease.

THE PRESIDENT inquired as to the chemical constitution of the volatile oils?

DR. H. P. MERRIMAN replied that many of the volatile oils were identical in chemical relations, but differed in physical properties. Such oils were *isomerides*. The essential oil of lemons, of bergamot, neroli, lavender, pepper, camomile, caraway, clover, etc., are isomerides of the oil of turpentine.

Oil of sassafras was an isomeride; whether or no of the turpentine group, he could not say. Oil of turpentine was a hydrocarbon, possessing the formula $C_{10}$ $H_{16}$.

DR. H. T. BYFORD was of the opinion that the oil of sassafras exerted its influence locally upon the alimentary canal and pelvic viscera, through which it was excreted, rather than upon the uterine nervous centres, as in the case of ergot. This would account for its popularity as an emmenagogue, mentioned by Dr. Sawyer. He had recently given one drop, combined with one-half grain of piperin, every three hours, for two weeks, in case of typhoid diarrhœa. Slight strangury, disappearing with the discontinuance of the drugs, was produced.

II. DR. CHARLES WARRINGTON EARLE presented for DR. JOSEPH HAVEN

A TERATOM, corresponding in development to the third month, and bearing an asserted resemblance to a pup.

The following history was read :

DR. HAVEN had attended the family of Mrs. H. for the past four years. During this time he had had occasion to notice that the younger daughter was a person unusually strong in her likes and dislikes, of a nervous temperament, slight build, yet a sensible, educated, and attractive girl.

On the eighth of September, 1885, this young lady, in company with her sister, called at his office to consult him with reference to her condition. He made the following entry in his case-book, as the result of her visit :

" Mrs. D., nineteen years old, married one and one-half years, always regular as to her courses up to July 21st, since then no show. Physical signs point to pregnancy in the sixth week."

A few days later he saw her again. She was nervous and higly excited—almost hysterical. She told him in an excited manner that a dog had jumped on her, and that she "hated dogs." She complained of pain in the abdomen, low down.

From that day until the 1st of November, Dr. Haven saw her several times. Each time she was threatened with miscarriage, and each time she declared she was positive she could never carry that child. Her husband and sister told him that, asleep or awake, her mind seemed to dwell continually upon that dog. That she daily wondered if the child would be marked. Mr. D. said that ever since he has known her she has been afraid of dogs ; she would always cross the street rather than meet one, and he has often jokingly refused to take her out with him, telling her, as an excuse, that they might see a dog, and she would make a scene.

On the night of November 1st, the husband roused DR. HAVEN, desiring him to go over and see his wife, thinking it to be only a repetition of former attacks. An examination proved that Mrs. D. was about to lose the contents of the uterus. She was flowing constantly. The os had dilated slightly and DR. HAVEN could just reach the presenting part. The history of the miscarriage was the usual one, and the result is seen in the specimen presented.

She insisted on seeing the fœtus, and declared it to be the image of the dog that had frightened her.

A general discussion upon the subject of maternal impressions followed.

III. PROFESSOR CHARLES WARRINGTON EARLE presented specimens from a case of artificial abortion. The fœtus corresponded in development to the fourth month of pregnancy, and was not decomposed. It was closely enveloped in the membranes, and entire absence of the *liquor amnii* was noticed.

Hæmorrhage into the placenta and decidua was not observed.

The following history of the case was read:

Mrs. F., American, has given birth to five children, the youngest twenty months old; labors always normal; has a history of anæmia for some months', if not years', standing; last menstruation ended May 20th, '85; in June, had a very slight discharge of blood; during the weeks following she would occasionally lose a small amount of blood, at other times there would be profuse hæmorrhage lasting twenty-four hours. She had at one time a white, sticky discharge, something like the albumen of an egg. Oct. 1st, began to flow constantly with some pain in back and sides, particularly the left. Was seen by PROFESSOR ST. JOHN, Oct. 12th, at which time he administered the usual styptics with rest. She continued to flow, with pain, for another week, when hæmorrhage was so severe and prostration so pronouneed, and with the suspicion of *placenta prævia* it was decided that temporizing means should cease. After consulting with PROFESSOR EARLE, it was decided to induce labour. A catheter was introduced and allowed to remain twenty-four hours, when pains came on and patient was delivered Oct. 17th, '85. During the entire period of gestation the woman could not detect the usual signs of her former pregnancies. She made a good recovery and menstruated Nov. 20th. There had been no discharge of water perceptible to the lady during the entire period of pregnancy.

DISCUSSION.

PROFESSOR W. W. JAGGARD thought PROFESSOR EARLE'S case was a typical example of the condition, technically termed *mummification.* The fœtus dies, and the fluid constituents of its body and envelopes are gradually resorbed. Mummification is usually observed in connection with twin pregnancies. One child is usually perfectly developed, while the other is converted into a mummy-like object.

*Maceration* and *mummification* of the fœtus are observed when the membranes are intact; *putrefaction*, after rupture and entrance of air into the uterine cavity. Professor Earle's case was probably not an example of that rare condition, abnormally small amount of amniotic fluid. There were no abnormal amniotic foldings, nor the fœto-amniotic bands described by SIMONART.

W. W. JAGGARD, M. D., *Editor*.

2330 Indiana Avenue.

